# Extra intravenous Ustekinumab reinduction is an effective optimization strategy for patients with refractory Crohn’s disease

**DOI:** 10.3389/fmed.2023.1105981

**Published:** 2023-07-24

**Authors:** Jiayin Yao, Xiang Peng, Yingkui Zhong, Tao Su, Adam Bihi, Junzhang Zhao, Tao Liu, Wei Wang, Pinjin Hu, Min Zhang, Min Zhi

**Affiliations:** ^1^Department of Gastroenterology, The Sixth Affiliated Hospital, Sun Yat-Sen University, Guangzhou, Guangdong Province, China; ^2^Guangdong Provincial Key Laboratory of Colorectal and Pelvic Floor Disease, The Sixth Affiliated Hospital, Sun Yat-Sen University, Guangzhou, Guangdong Province, China

**Keywords:** Crohn’s disease, Ustekinumab, optimization algorithm, intravenous reinduction, endoscopic remission

## Abstract

**Objectives:**

Ustekinumab (UST) optimization strategies, including shortening intervals and intravenous reinduction, should be administered to patients with partial or loss of respond. Evidence comparing these types of optimization treatments is limited. We evaluated the efficacy and safety of weight-based UST intravenous reinduction in patients with refractory Crohn’s disease (CD).

**Methods:**

This was a single-center retrospective observational study. Optimization strategies were designed for patients showing partial or loss of response to standardized UST therapy. Clinical, biochemical, and endoscopic response and remission rate were determined by Crohn’s disease activity index (CDAI), C-reactive protein (CRP) levels, and SES-CD evaluation. UST trough concentrations were detected and adverse events were recorded.

**Results:**

A total of 128 patients receiving UST optimization therapies were included, with 105 patients administered shortening intervals of q8w or q4w, and 23 receiving intravenous reinduction followed by subcutaneous q8w or q4w. The follow-up duration for the shortening interval and reinduction cohorts were 15.0 (10.0, 31.0) and 23.0 (13.0, 70.0) weeks, respectively. A significant CDAI delta variation pre-and post-treatment could be found between groups [17.0 (−4.4, 65.9) vs. 69.0(10.7, 151.0), *p* = 0.013]. the trough concentration of UST increased [2.5 (1.3, 5.3) vs. 1.1 (0.5, 2.3), *p* = 0.001] after intravenous reinduction. Clinical and endoscopic remission were achieved in 69.6 and 31.8% of patients in the intravenous reinduction cohort, and 62.9 and 22.2% of patients in the shortening interval cohort, respectively. No significant difference was found between groups regarding safety.

**Conclusion:**

Intravenous reinduction brought about favorable recapture of clinical and endoscopic remission, and should have significant priority over the strategy of merely shortening drug intervals, which should be launched before switching to other biologics targeting different inflammatory pathways.

**Clinical Trial Registration:** identifier NCT04923100. https://classic.clinicaltrials.gov/ct2/show/NCT04923100?id=04923100&draw=2&rank=1

## Introduction

Ustekinumab (UST), a human monoclonal antibody targeting the p40 subunit shared by interleukin (IL)-12 and IL-23, was approved for the treatment of moderate-to-severe Crohn’s disease (CD) in American in 2016, and China in 2020 (1, 2). With this four-year gap in approval, real-world data concerning efficacy and optimization of UST are limited in the Chinese population. We first demonstrated favorable clinical and endoscopic remission rates in patients who were administrated UST as a second-line biologic after loss of respond to anti-TNF agents in 2021, right after the approval of UST in treating CD in China (3). Along with the dramatic drop in the cost and increasing number of patients receiving UST treatment, non-complete response and secondary loss of response to UST cannot be ignored, especially in patients with refractory CD. Multiple observational studies have reported that loss of response rates to UST ranged from 27 to 34% (4, 5), which draws our focus to UST optimization strategy.

For patients who fail to respond to standardized UST treatment, optimization strategies should be considered before switching to biologics targeting different inflammatory pathways (6). UNITI trials have demonstrated that shortening the dosing intervals from every 12 weeks (q12w) to q8w help recapture clinical remission (7–9). A proportion of patients with refractory CD respond poorly even on the already shortening intervals of q8w or q4w (10). Intravenous UST reinduction should be launched, which may be an optimal optimization strategy, although it is supported by few studies (11–13). In this study, we aimed to evaluate the efficacy of intravenous UST reinduction in patients with refractory CD in a real-world setting, with a particular focus on the advantages of intravenous UST reinduction as compared with only shortening drug intervals.

## Materials and methods

### Study design and patient population

In this retrospective observational cohort study, patients with confirmed diagnosis of CD and who underwent UST treatment at the Sixth Affiliated Hospital of Sun Yat-Sen University from 1 March 2020 to 30 September 2022 were enrolled. Clinical data were collected from hospital’s digital record. The diagnosis of CD was made according to the widely accepted criteria (14, 15) with clinical symptoms, biomarker changes, endoscopic findings, and imaging manifestations comprehensively considered. Disease location and behavior were documented according to the Montreal classification (16). This study was approved by the Ethics Committee of Sun Yat-Sen Sixth Affiliated Hospital (2021ZSLYEC-066) and registered online (NCT04923100). Informed consent was waived due to the retrospective design of the study.

### UST escalation and reinduction

All the patients with CD and treated with scheduled UST therapy were recommended to return for short-term efficacy reevaluation at week 16 after UST initiation, the very timepoint before the third infusion of UST. Patients who successfully achieved clinical remission would be administered the subcutaneous UST q12w, and those who failed to achieve clinical remission would be administered optimization strategies including shortening intervals of q8w or q4w, or weight-based intravenous reinduction followed by subcutaneous q8w or q4w at the discretion of clinicians. Patients were followed up every 3 months to evaluate both clinical and serological remission. When partial response or loss of response to UST were found during the follow-up period, optimization therapy was given at the discretion of clinicians. Endoscopy examination was recommended at week 16 and 1 year after UST initiation. Serum trough concentrations of UST were recorded immediately before and 8 weeks after optimization.

### Outcomes and definitions

The primary outcome was clinical remission at 3 months after optimization treatment. Secondary outcomes included clinical, biochemical, and endoscopic response and remission, and safety assessment at 1 year after UST initiation. Clinical remission was defined as a Crohn’s disease activity index (CDAI) of <150, and clinical response was defined as a decrease of CDAI of >70 from the baseline value (17). Endoscopic remission was defined as a simplified endoscopic score for Crohn’s disease (SES-CD) of ≤2, and the endoscopic response was defined as a decrease in SES-CD of >50% from the baseline value (18). Rutgeerts (19) scores were evaluated for patients who had undergone previous colon surgeries. Endoscopic remission was defined as a Rutgeerts score of ≤ i1, whereas endoscopic response was defined as a reduction in one grade from the baseline score (20, 21). C-reactive protein (CRP) normalization was defined as a CRP level of <4 mg/L. Loss of response was defined as a patient who achieved none of the clinical, biochemical, or endoscopic response. Partial response referred to a patient who achieved at least one of the clinical, biochemical, or endoscopic response (14, 15). Adverse events were recorded to evaluate the safety profile of UST optimization.

### Statistical analysis

Continuous variables are presented as the mean with standard error of the mean (SEM) or as the median with interquartile range (IQR) according to data distribution. Categorical variables are presented as proportions or rates. Continuous variables were analyzed using the t-test or Mann–Whitney test, as appropriate. Comparison of rates was conducted using Chi-squared test. Statistical significance was set at two-sided *p* < 0.05. IBM SPSS software (version 20.0, IBM Corp., Armonk, N.Y., United States) was used for data analysis.

## Results

### Patients’ characteristics

**Figure 1:**
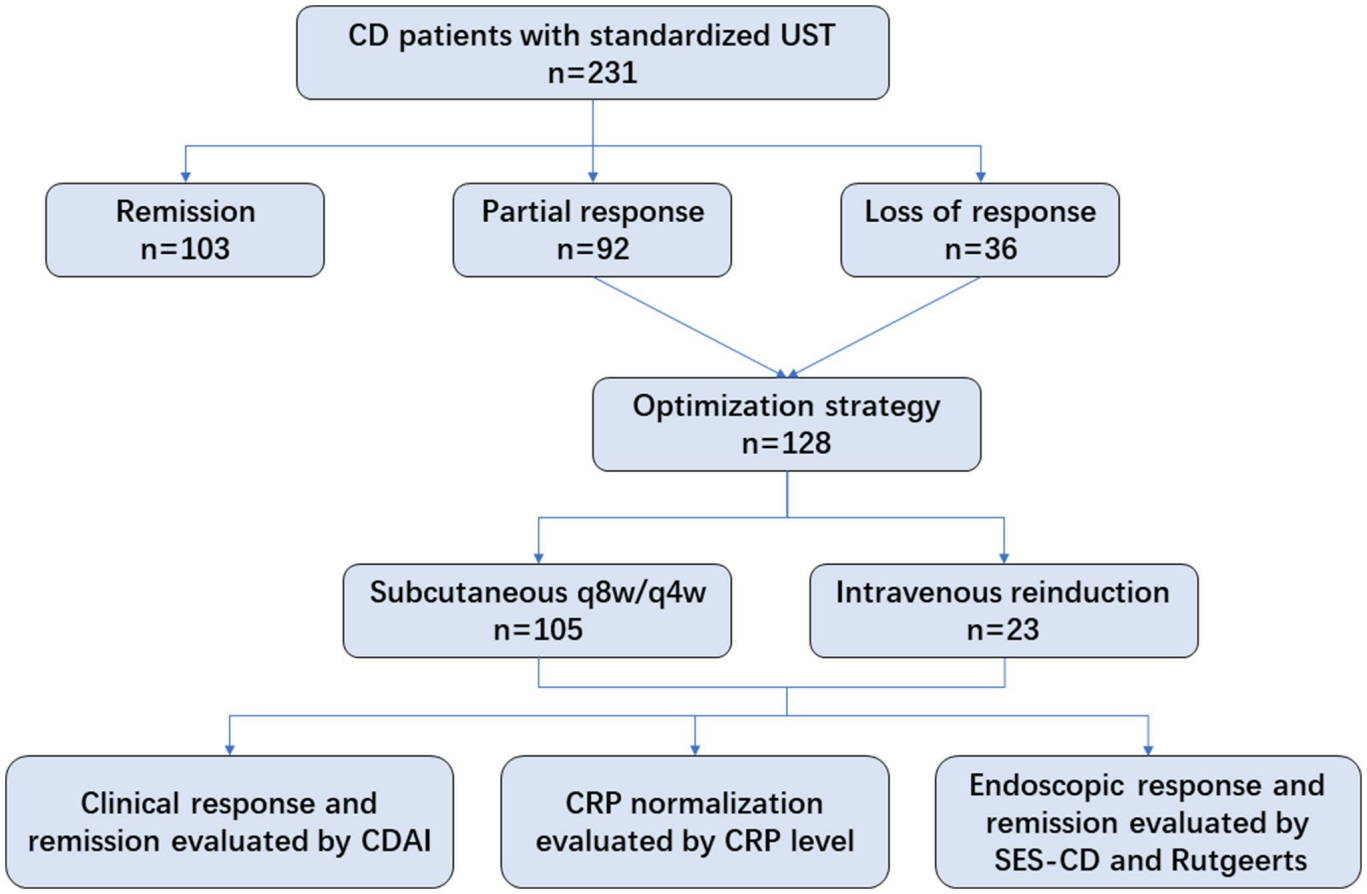
Flow chart of this study.

A total of 128 eligible patients receiving UST optimization therapies were included, of whom 105 patients were administered shortening intervals of q8w (*n* = 100) or q4w (*n* = 5), and 23 underwent intravenous reinduction followed by subcutaneous q8w (*n* = 20) or q4w (*n* = 3). Regarding the indications for optimization strategies, 71.9% of patients showed partial response, and 28.1% showed loss of response to UST evaluated at week 16 (Figure 1). Patients’ baseline characteristics are summarized in Table 1.

**Table 1:** Patients’ characteristics in different cohorts.

Variables	Patients with q8w/q4w SC (*n* = 105)	Patients with IV reinduction (*n* = 23)	*p* value
Male, *n* (%)	85 (81.0)	18 (78.3)	0.769
Age at diagnosis, [years, Median (IQR)]	28.0 (22.5,39.0)	28.0 (24.0,33.0)	0.978
Disease duration, [years, Median (IQR)]	2.5 (1.0,6.0)	4.0 (1.0,10.0)	0.136
Montreal classification			0.571
Age, *n* (%)			
A1(≤16 years)	3 (2.9)	0 (0)	
A2(17–40 years)	80 (76.2)	20 (87.0)	
A3(>40 years)	22 (20.9)	3 (13.0)	
Disease behavior, *n* (%)			0.053
B1(non-stricturing, non-penetrating)	45 (42.9)	15 (65.2)	
B2(stricturing)	41 (39.0)	6 (26.1)	
B3(penetrating)	19 (18.1)	2 (8.7)	
Disease location, *n* (%)			0.312
L1(ileal)	36 (34.3)	7 (30.4)	
L2(colonic)	2 (1.9)	1 (4.3)	
L3(ileocolonic)	65 (61.9)	15 (65.2)	
L4(upper GI)	2 (1.9)	1 (4.3)	
Previous intestinal surgery, *n* (%)	34 (32.4)	3 (13.0)	0.065
Previous perianal surgery, *n* (%)	47 (44.8)	14 (60.9)	0.163
Previous therapies, *n* (%)			
Mesalamine	70 (66.7)	18 (78.3)	0.279
Immunosuppressants^1^	68 (64.8)	20 (87.0)	0.038
Steroids	46 (43.8)	12 (52.2)	0.467
Biologics	54 (51.4)	21 (91.3)	0.007
Number of previous biologics			0.007
1	39 (37.1)	18 (78.3)	
2	12 (11.4)	3 (13.0)	
≥3	3 (2.9)	0 (0)	
Concomitant therapy at initiation			
Steroids	7 (6.7)	6 (26.1)	0.005
Immunosuppressants	6 (5.7)	0 (0)	0.242
Extraintestinal manifestation, *n* (%)	29 (27.6)	4 (17.4)	0.312
Baseline CRP, mean ± S.D.E	17.0 ± 2.7	19.7 ± 5.2	0.662
Baseline CDAI, mean ± SD	144.6 ± 7.5	156.7 ± 15.9	0.497

1 Immunosuppressants includes thiopurines, methotrexate, cyclophosphane, and thalidomide.

IQR, interquartile range; SC, subcutaneous; IV, intravenous; GI, gastrointestinal; IQR, interquartile range; IV: intravenous; q8/4w: every 8/4 weeks; SC, subcutaneous; UST, ustekinumab; CRP, C-reactive protein; CDAI, Crohn’s disease activity index; S.D.E, standard error.

### Clinical and biochemical outcomes

Despite the switch to adalimumab in three patients due to failure in response and in two patients due to UST allergy, 96.1% of patients continued UST maintenance therapy. The median (IQR) time interval from initiation of UST and optimization was 17.0 (8.0, 21.5) weeks and 16.0 (9.0, 18.0) weeks in the shortening interval cohort and the reinduction cohort, respectively. The follow-up duration for the shortening interval and reinduction cohorts were 15.0 (10.0, 31.0) and 23.0 (13.0, 70.0) weeks, respectively. CDAI scores decreased significantly in the reinduction cohort (*p* < 0.05) (Table 2). The clinical and biochemical remission rates were 62.9 and 50.5% in the shortening interval cohort, 69.6 and 56.5% in the reinduction cohort, respectively.

**Table 2:** Evaluations of CRP and CDAI before and after optimization in the two cohorts.

Variables	Patients with q8w/q4w SC (*n* = 105)		Patients with IV reinduction (*n* = 23)		*P* value
	median (IQR)	Time to assessment [weeks, median (range)]	median (IQR)	Time to assessment [weeks, median (range)]	
CRP delta variation	0.4 (−0.6, 7.2)	8.0 (8.0, 16.0)	3.2(−0.8, 8.4)	8.0 (8.0, 8.0)	0.225
CDAI delta variation	17.0 (−4.4, 65.9)	8.0 (8.0, 16.0)	69.0 (10.7, 151.0)	8.0 (8.0, 16.0)	0.013

IQR, interquartile range; q8/4w, every 8/4 weeks; SC, subcutaneous; IV, intravenous; CRP, C-reactive protein; CDAI, Crohn’s disease activity index; CRP delta variation: deviation of CRP between pre-and post-treatment; CDAI delta variation: deviation of CDAI between pre-and post-treatment.

### Endoscopic outcomes

A total of 94 patients underwent paired endoscopy before and after treatment optimization, of which 89 (67 in the q8w cohort and 22 in the reinduction cohort) and 5 (all in the q8w cohort) patients were evaluated by SES-CD and by Rutgeerts scores, respectively. Endoscopic remission rates were 22.2 and 31.8% in the shortening interval and reinduction cohorts, respectively. Patients in the reinduction cohort appeared to achieve a higher rate of endoscopic remission, but without a statistical significance yet (Table 3).

**Table 3:** Biochemical, clinical, and endoscopic evaluations of patients in different cohorts.

Variables	Patients with q8w/q4w SC (*n* = 105)	Patients with IV reinduction (*n* = 23)	*p* value
CRP normalization, n/n (%)	53/105 (50.5)	13/23 (56.5)	0.599
Clinical response, n/n (%)	67/105 (63.8)	18/23 (78.3)	0.184
Clinical remission, n/n (%)	66/105 (62.9)	16/23 (69.6)	0.544
Endoscopic response, n/n (%)	32/72 (44.4)	12/22 (54.5)	0.406
Endoscopic remission, n/n (%)	16/72 (22.2)	7/22 (31.8)	0.360

CRP, C-reactive protein; IV, intravenous; q8/4w, every 8/4 weeks; SC, subcutaneous; UST: ustekinumab.

### Multivariable analysis

We further conducted a multivariable regression analysis to analyze the differences between the two cohorts thoroughly. The CRP delta variation, which was defined as the deviation of CRP between pre-and post-treatment, was found to be different between groups, with statistical significance noted (*p* < 0.05) (see Table 4).

**Table 4:** Multivariate regression analyses of variables in the two cohorts.

Variables	OR	95% CI	*p* value
CRP normalization	0.751	0.172–3.284	0.704
Clinical response	0.047	0.002–1.082	0.056
Clinical remission	11.761	0.555–249.096	0.114
Endoscopic response	1.287	0.246–6.741	0.765
Endoscopic remission	0.790	0.187–3.327	0.748
CRP delta variation	1.018	1.003–1.033	0.019
CDAI delta variation	0.996	0.988–1.003	0.286

CRP, C-reactive protein; OR, odds ratio; CI, confidence interval; CRP delta variation: deviation of CRP between pre-and post-treatment; CDAI delta variation: deviation of CDAI between pre-and post-treatment.

### Trough concentration changes

**Figure 2:**
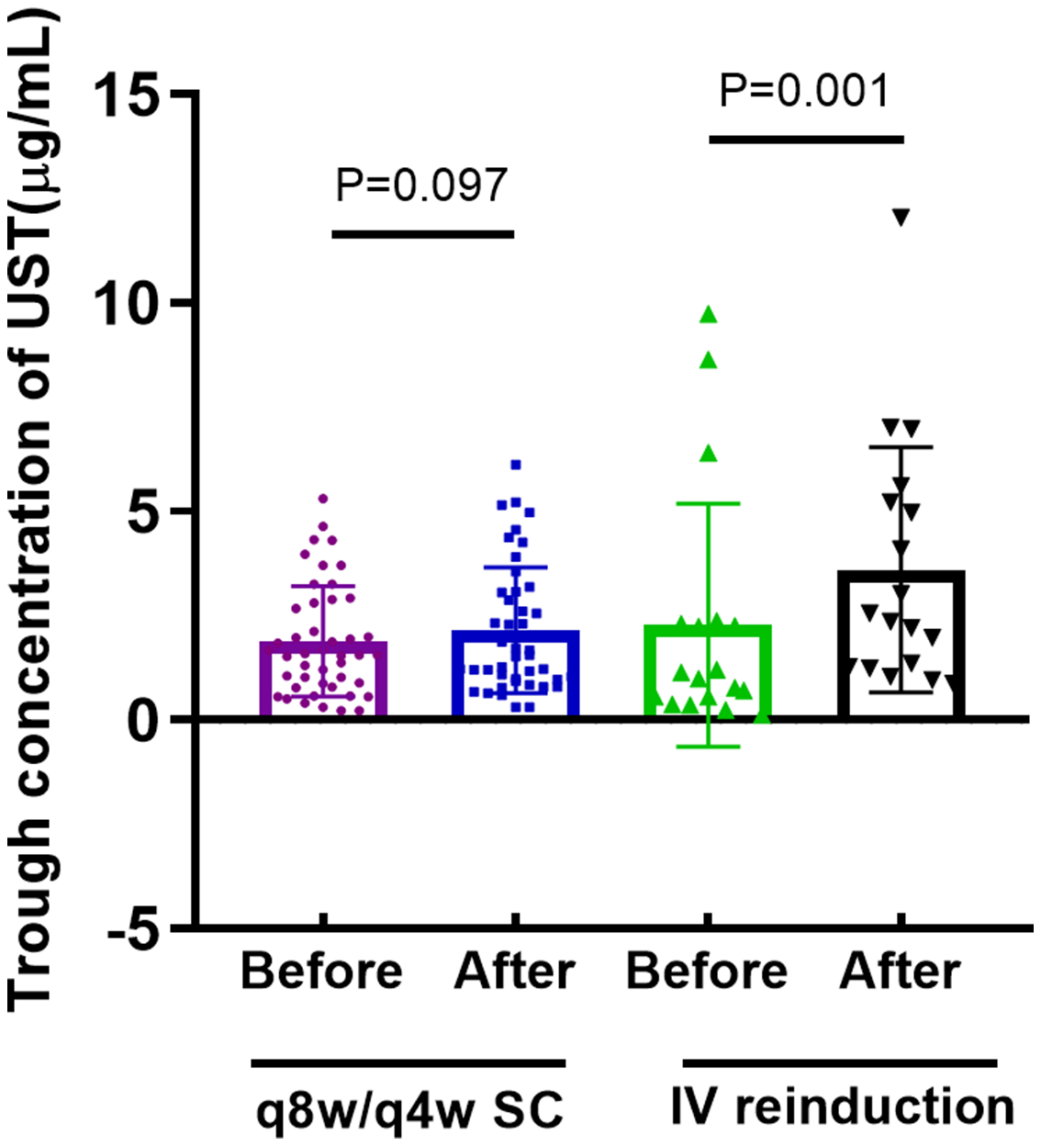
Comparison of trough concentration of UST before and after optimization strategy. (q8w or q4w SC: every-8-week or every-4-week subcutaneous dosing; IV reinduction: intravenous reinduction on a weight-based dosing regimen; q8/4w or q4w SC: every-8-week or every-4-week subcutaneous dosing; UST: ustekinumab).

UST trough concentrations, measured before and 8 weeks after optimization, were retrieved in 45 and 11 patients in the shortening interval and reinduction cohorts, respectively. Patients in the intravenous reinduction group showed a significant increase in UST trough concentrations [1.1 (0.5, 2.3) vs. 2.5 (1.3, 5.3), *p* = 0.001] after optimization (Figure 2).

### Safety evaluation

Two patients (one in shortening interval group and the other in IV reinduction group) showed allergy to intravenous UST and switched to another biologics, and three patients (all in shortening interval group) complained of pruritis, which was relieved after anaphylaxis treatment. The total rate of adverse effect was 3.8 and 4.3% in shortening interval group and IV reinduction group, respectively (*p* > 0.05). No severe adverse events were observed throughout our study, and no significant difference could be found between groups (see Table 5).

**Table 5:** Treatment safety evaluation between grous.

Adverse effect	Patients with q8w/q4w SC (*n* = 105)	Patients with IV reinduction (*n* = 23)	*p* value
Allergy (n/n,%)	1/105 (1.0)	1/23 (4.3)	0.236
Pruritis (n/n,%)	3/105 (2.9)	0/23 (0)	0.414
Total (n/n,%)	4/105 (3.8)	1/23 (4.3)	0.904

## Discussion

In our results, UST optimizing strategies enabled more than half of the patients to recapture clinical remission after initially poor or loss of response to UST, indicating the potential benefits gained from UST-optimized treatment before switching to other biologics. Weight-based intravenous reinduction seems to merit priority in remission recapture compared with merely shortening drug intervals.

Dosing escalation with shortening intervals of q8w or q4w is reportedly effective in regaining clinical and endoscopic responses (22, 23). However, a proportion of patients still show poor responses on the already q8w and q4w drug intervals, which calls for intravenous reinduction as a rescue treatment. To date, published studies evaluating the efficacy and safety of extra weight-based intravenous doses of UST are scarce (24, 25). A recent study including 65 patients has demonstrated that 31% of patients achieved clinical remission off corticosteroids after UST intravenous reinduction (26). Bermejo F et al. from Spain performed a retrospective observational study including 53 patients from 13 centers, and revealed that clinical remission rates at week 8 and 16 after reinduction were 49.0 and 43.3%, respectively (11). Another study reported that the clinical remission rates after reinduction at week 8, 20 and 52 were 37, 56 and 45%, respectively, in 31 CD patients with a secondary loss of response to UST (12). Other real-world cohorts with relatively small sample sizes from America (*n* = 13) (13), Israel (*n* = 30) (27), and the Netherlands (*n* = 7) (28) demonstrated response rates ranging from 40 to 50%. Most of these studies reported clinical changes only, without endoscopic data and trough concentrations of UST considered, which limited their values in clinical application.

We reported a higher clinical remission rate than previously reported in the published literatures, which could be attributed to the following reasons. First, patients were routinely followed up and intensively evaluated at an early timepoint of week 16, which enables early recognition of partial or loss of response to UST, and timely launch of optimized treatment. It eventually led to a higher rate of remission recapture and better outcomes. Moreover, we determined clinical and biochemical response by evaluating CDAI scores and CRP levels at a median of 8 weeks, which is a relatively short duration. Since short-term response was obtained more easily than long-term maintenance, undoubtedly, a prospective large-sample trial evaluating the long-term efficacy is urgently needed. The POWER study (NCT03782376), an ongoing randomized controlled trial, will provide sufficient evidence on the efficacy and safety profile of UST intravenous reinduction and will fill in the blank to aid clinical practice.

Of note, the groups were homogeneous in the majority of characteristics such as age, gender, Montreal Classifications, and previous surgery, but heterogeneous in both previous and concomitant treatment. Patients in the reinduction cohort were observed to have higher rates of previous biologics treatment and steroid concomitant therapy, indicating the refractory characteristics of disease phenotype. Compared with those in shortening interval cohort, patients in the intravenous reinduction cohort manifested a significant reduction in CDAI scores before and after optimization treatment, along with a significant increase in UST trough levels. Moreover, higher rates of CRP normalization, clinical response and remission, and endoscopic response and remission were observed in patients in reinduction cohort, which indicated a better performance of reinduction over shortening interval despite the absence of statistical significance. Our previous study demonstrated the drug exposure-response relationship in patients receiving UST therapy (3). UST trough concentration was closely associated with clinical and endoscopic remission rates (29), with underlying mechanisms focusing on the UST volume distribution (30, 31). Patients with active disease activity always manifest higher levels of pro-inflammatory cytokines IL-12/23 detected, calling for more binding with UST, and resulting in lower drug concentrations (32). As a result, intravenous reinduction with weight-based dosing elevates the trough concentration (7, 33), leading to recapture of response. Regarding tissue UST concentration, there are only two relevant studies to date. A real-world analysis conducted in patients with ulcerative colitis confirmed a drug exposure-response relationship, but denied the utility of measuring tissue’s drug level, given its strong correlation with serum exposure (34). The other study demonstrated a strong correlation between serum and tissue UST levels in patients with CD and concluded that UST serum levels were more indicative of biochemical response (31). We detected the serum level, instead of tissue, UST levels in our clinical routine due to its less-invasiveness, convenience, and repeatability.

As for safety, UST is supposed to be a safer biologic agent than anti-TNF agents, with fewer adverse effects reported (35, 36). A total of 96.1% patients with UST optimization therapy remained on scheduled maintenance therapy without severe adverse effects observed. The patient-reported adverse effects rate was 3.8 and 4.3% in shortening interval group and intravenous reinduction group, respectively, which were similar to those reported by IM-UNITI trial (37). Undoubtedly, it will add evidence on the safety profile of UST, even in the optimization therapy.

This study had some limitations. Selection bias should be comprehensively considered due to its retrospective design. The single-center data source may limit the reliability of the study in clinical application. However, our complete data and rigorous definition help compensate for this shortcoming. Notably, UST has been available in the Chinese market for only 2 years, and intravenous reinduction is narrowly investigated worldwide. Therefore, our real-world data, undoubtedly, help support the profound clinical utility of UST optimization therapy before biologic switching.

**Figure 3:**
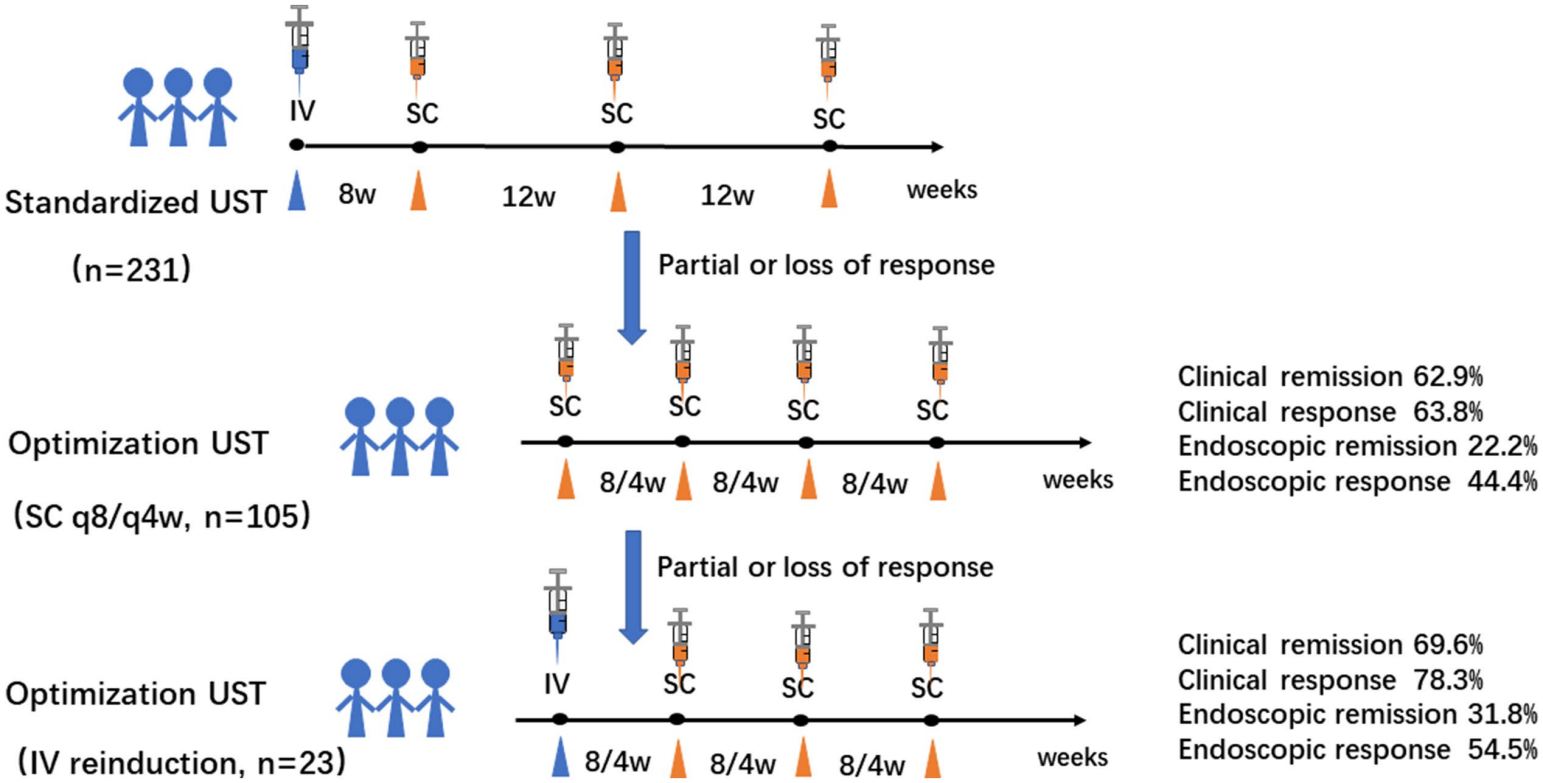
Graphic summary of the study. Intravenous reinduction of UST followed by subcutaneous q8w or q4w is a favorable rescue optimization strategy for refractory CD patients with clinical and endoscopic remission of 69.6 and 31.8%, respectively. (CD: Crohn’s disease; q8/4w: every 8/4 weeks; UST: UST: ustekinumab).

In conclusion, patients with refractory CD who fail to respond to UST may gain benefits from intravenous reinduction in terms of remission recapture with a favorable safety profile (Figure 3). Prospective trials with larger sample-sizes are warranted to evaluate the efficacy of different types of UST optimization in-depth.

## Data availability statement

The raw data supporting the conclusions of this article will be made available by the authors, without undue reservation.

## Ethics statement

The studies involving human participants were reviewed and approved by the Ethics Committee of Sun Yat-Sen University (2021ZSLYEC-066) and by the Clinical Trial Registry (NCT04923100). Written informed consent for participation was not required for this study in accordance with the national legislation and the institutional requirements.

## Author contributions

JY, XP, YZ, TS, AB, JZ, TL, WW, PH, MZha, and MZhi contributed to the study conception and design. Material preparation, data collection and analysis, manuscript drafting were performed by JY, XP, and YZ. Acquisition and analysis of data were performed by TS, AB, JZ, TL, WW, and PH. Critical revision of the manuscript was performed by MZha and MZhi. Final approval of the version to be submitted was performed by MZhi. All authors contributed to the article and approved the submitted version.

## Funding

This study was supported by the National Natural Science Foundation of China [81900490, 81670477 and 82270544], and Yong Teacher Foundation of Sun Yat-Sen University [22qntd3604], Project 1010 of Sixth Affiliated Hospital of Sun Yat-Sen University [1010PY(2020)-55], Qingfeng Scientific Research Fund of the China Crohn’s & Colitis Foundation (CCCF-QF-2022A53-2 and CCCF-QF-2022B43-14) and the program of Guangdong Provincial Clinical Research Center for Digestive Diseases (2020B1111170004).

## Conflict of interest

The authors declare that the research was conducted in the absence of any commercial or financial relationships that could be construed as a potential conflict of interest.

## Publisher’s note

All claims expressed in this article are solely those of the authors and do not necessarily represent those of their affiliated organizations, or those of the publisher, the editors and the reviewers. Any product that may be evaluated in this article, or claim that may be made by its manufacturer, is not guaranteed or endorsed by the publisher.
